# Long term outcomes among African stroke survivors: 4 years follow up data from the CogFAST—Nigeria Study

**DOI:** 10.3389/fstro.2025.1586814

**Published:** 2025-12-19

**Authors:** Gabriel Ogunde, Joshua Akinyemi, Louise Allan, Mayowa Owolabi, Adesola Ogunniyi, Rajesh N. Kalaria, Rufus Akinyemi

**Affiliations:** 1 Neuroscience and Ageing Research Unit, Institute for Advanced Medical Research and Training, College of Medicine, University of Ibadan, Ibadan, Nigeria; 2 Department of Epidemiology and Medical Statistics, Faculty of Public Health, College of Medicine, University of Ibadan, Ibadan, Nigeria; 3 Epidemiology and Biostatistics Research Unit, Institute for Advanced Medical Research and Training (IAMRAT), College of Medicine, University of Ibadan, Ibadan, Nigeria; 4 College of Medicine and Health, University of Exeter, Exeter, United Kingdom; 5 College of Medicine, University of Ibadan and University College Hospital, Ibadan, Nigeria; 6 Department of Neurology, University College Hospital, Ibadan, Nigeria; 7 Translational and Clinical Research Institute, Newcastle University, Newcastle upon Tyne, United Kingdom

**Keywords:** stroke, cognitive impairment, mortality, functional dependence, parametric, depression, caregiver burden

## Abstract

**Introduction:**

Although stroke is recognized as a chronic condition, estimates of different long-term outcomes after stroke are lacking in Africa. This study aimed to explore the profile, trajectory and determinants of long-term outcomes up to 4 years in a cohort of African stroke survivors.

**Method:**

The data analyzed were collected in a longitudinal study of stroke survivors who were prospectively recruited into the CogFAST-Nigeria Study from two specialist hospitals in Nigeria. Subjects with subarachnoid hemorrhage, co-morbid psychiatric or neurologic illness, or any systemic disease that could impair cognition were excluded from the study. Cognition was assessed using the Vascular Neuropsychological Battery, depression with the Geriatric Depression Scale—short form, and functional performance with the Barthel Index. Weibull survival model, generalized estimating equation and linear mixed models were used to identify the predictors of mortality, cognitive impairment, functional performance, and caregiver burden respectively.

**Result:**

Of the 253 stroke survivors that were recruited into the study, 157 (59.7%) were males while the overall mean age was 60.2 ± 9.8 years.The proportions of those with cognitive impairment were 126/251 (50.2%) at 3 months after stroke, 69/160 (43.1%), and 12/36 (33.3%) at 1^st^ and 4^th^ year respectively, while the proportion of those with depression was 39.3% at 3 months post-stroke, 35.2%, and 36.1% at year 1 and 4 respectively. Cumulative Mortality increased from 13.8% (95% CI = 10.08–18.63) at 9 months post-stroke to 45.3% (95% CI = 39.42–51.6) at 4 years follow-up. The only factor associated with mortality after adjusting for ethnicity was working as an artisan (aHR = 2.22; 95% CI = 1.77–4.02). History of previous stroke increased the likelihood of functional dependency (OR = 2.17; 95% CI = 1.19–3.95). Meanwhile, higher education (OR = 0.05; 95% CI = 0.02–0.16) protected against cognitive impairment while previous stroke (OR = 2.17; 95% CI = 1.19–3.95;) and higher caregiver burden (OR = 1.02; 95% CI = 1.01–1.02) were associated with increased risk.

**Conclusion:**

Improving stroke treatment and rehabilitation is crucial, especially for those with prior stroke, as it strongly predicts poor functional and cognitive outcomes.

## Highlights

- Cumulative mortality probability increased from 13.8% at 9 months post-stroke to 45.3% at four years.
- After adjusting for ethnicity, being an artisan increased the risk of mortality (aHR = 2.16)
- Prior stroke (aOR = 2.16) and age at stroke recurrence (aOR = 1.04) increased the risk of functional dependency among the stroke survivors.

## 1 Introduction

Stroke is a leading cause of death and disability worldwide (World Stroke Organization, 2024), and it is a looming epidemic of the twenty-first century (Akinyemi et al., 2021; Donkor, 2018; Owolabi et al., 2015). Globally, 1 in 4 adults over the age of twenty-five will have a stroke in their lifetime, about 13.7 million people will have their first stroke, while 5.5 million will die as a result (Donkor, 2018). The incidence of stroke has declined or plateaued in high income countries but has increased in low-and middle-income countries (Wang et al., 2015). When compared to other populations, Africans have a greater risk of stroke, considerably worse outcomes, and a high burden of non-motor post-stroke co-morbidities (Sarfo et al., 2018).

The prevalence of stroke in Africa is up to 1460 per 100,000 persons, its incidence is up to 316 per 100,000 person-years while case fatality rate is up to 84% at 3 years (Akinyemi et al., 2021). Stroke case-fatality rates in sub-Saharan Africa (SSA) are the highest in the world ranging from 21 to 47% (Akinyemi et al., 2021; Okekunle et al., 2023). One month stroke case-fatality rate in Africa is high-−24.45% with higher estimates of 27.57% among females (Okekunle et al., 2023). According to projections, the adoption of western lifestyles, greater urbanization, a lack of risk knowledge, and poor healthcare infrastructure will all contribute to an increase in the burden of stroke in Sub-Saharan Africa in the next decades (BeLue et al., 2009).

Stroke requires long-term management (GBD 2019 Stroke Collaborators, 2021; Kim et al., 2020; Hill et al., 2022); however, estimates of different outcomes after stroke in the long term, beyond one year, are lacking, with most of the existing data on stroke outcomes being limited to short-term cohort studies with limited follow-up (usually up to 1 year), as well as focusing on disability alone or only a few outcome measures (Wolfe et al., 2011; Stolwyk et al., 2021; Lo et al., 2022). The present study, therefore, reports findings from a 4 year follow up of the trajectory and predictors of long-term outcomes (mortality, functional dependency, caregiver burden and cognitive impairment) among stroke survivors participating in the CogFAST- Nigeria Study, a longitudinal study of stroke survivors who were prospectively recruited from two specialist hospitals in Nigeria (Akinyemi et al., 2014).

## 2 Materials and methods

The study population consists of stroke survivors (aged >35 years) who were recruited between July 2010 and June 2012 and were followed up longitudinally at two different specialist hospitals: the University College Hospital (UCH), Ibadan and the Federal Medical Centre (FMC), Abeokuta both in Nigeria. Patients were evaluated for neurological impairment, motor disability, and activities of daily living using the modified Rankin Scale (Wilson et al., 2002), stroke severity using the Stroke Levity Score (Owolabi and Platz, 2008), functional performance (the Barthel Index) (Obembe and Oluwatosin, 2012) caregiver burden (the Caregiver Strain Index) (Ogunlana et al., 2014), and depressive symptoms using the Centre for Epidemiologic Studies Depression Scale (Yesavage et al., 1982). Details of these evaluations have been published elsewhere (Akinyemi et al., 2014).

### 2.1 Study exclusion criteria

We excluded stroke survivors with age less than 35 years, subarachnoid hemorrhage, significant physical illness and motor impairment that precluded paper and computer-based neuropsychological evaluation (e.g., visual impairment, moderate-severe aphasia, hemiparesis affecting the dexterous hand, MRC power grade <3), any co-morbid psychiatric or neurologic illness, and any systemic disease that could impair cognition e.g., chronic liver disease, chronic kidney disease. We excluded individuals with early onset or young-onset stroke below 35 years of age because the focus of the study was originally on cognitive dysfunction associated with stroke which is primarily an aging—associated disease condition. Also, sub-arachnoid hemorrhage (SAH) cases were excluded as they were relatively few, and SAH does not primarily affect brain parenchyma. Specifically, we included patients with either spontaneous intracerebral hemorrhage or cerebral infarction at baseline. The specificity of brain tissue damage (vascular brain injury) was considered necessary for us to examine the impact on cognitive function.

### 2.2 Study outcome measures

Scientific data that support the effectiveness of rehabilitation programs and interventions for stroke patients mostly focus on post-stroke outcomes such as mortality, functional dependency, cognitive function, depression etc.

#### 2.2.1 Mortality

Mortality following stroke in this study was determined through two complementary approaches: death certificates or reported death via telephone conversation with the caregiver. Death certificates, obtained from official registries, provided a formal record of cause and date of death, ensuring accuracy and standardization. For cases where death certificates were unavailable, family members/caregivers to the stroke survivor were contacted to determine if the participant was alive or not (Akinyemi et al., 2014). During the call, questions were asked about the living status of the patient, date of death (if the patient had died), and possible cause of death, to determine if the death was stroke-related or not. In many instances, however, the living relatives were frugal with information as they did not want to be reminded about the loss of their loved ones.

#### 2.2.2 Functional dependency

In this study, the Barthel Index was used to measure functional dependency among stroke survivors. With a maximum score of 20, the Barthel Scale/Index (BI) is a 10-item scale that is used to assess performance of daily activities (ADL) with a higher score reflecting greater ability to function independently following hospital discharge. Scores ranging from 0 to 14 were classified as “dependent” and assigned a value of one (1), while scores of 15 to 20 were categorized as “independent” and assigned a value of zero (0). The scale has been validated (Obembe and Oluwatosin, 2012) in southwest Nigeria where this study was conducted the Yorubas.

#### 2.2.3 Caregiver burden

Caregiver burden was assessed using the Caregiver Strain Index (CSI). The CSI is a 13-item scale that records responses to activities and tasks to help the stroke survivor. They are then graded with a maximum possible score of 13. A score of 7 or more indicates a greater level of stress. The CSI is widely used in Nigeria and reported to have high reliability (Sullivan, 2002; Ogunlana et al., 2014).

#### 2.2.4 Cognitive assessment

The Community Screening Instrument for Dementia (CSID)—cognitive part, the Mini-Mental State Examination (MMSE), and the Vascular Neuropsychological Battery (V-NB) (Akinyemi et al., 2014; Hachinski et al., 2006) made up the cognitive assessment tools. The V-NB is a battery of tests that assesses cognition in specific domains, whereas the CSID and the MMSE are broad tests of cognitive functioning. The V-NB, which was previously used in the CogFAST-NG study, comprised of many validated test items measuring particular cognitive domains (executive function, memory/learning, language, visuospatial/visuoconstructive skills). The full details of cognitive evaluation of the cohort and how cognitive diagnosis was made on each study subject have been fully described in an earlier report on the cohort (Akinyemi et al., 2014). Essentially, to make a cognitive diagnosis on a subject, all available datasets including cognitive scores, functionality and disability scores (the Barthel Index and modified Rankin score) coupled with the physician's assessment were assembled and discussed by the research team for consensus diagnosis. Functional impairment was defined as a Barthel Index score <75 (Uyttenboogaart et al., 2005). Final cognitive categorization was based on the VCI criteria proposed by the American Stroke Association/American Heart Association VCI Guidelines (Gorelick et al., 2011) and the DSM IV criteria (American Psychiatric Association, 1994).

### 2.3 Statistical analysis

Individual-level longitudinal data were analyzed. First, descriptive analysis was done to summarize the study participants' (stroke survivors) background characteristics. It was also used to summarize the study outcomes (such as functional dependency and cognitive function). In addition, the Kaplan Meier curve was employed to describe survival rates over the four-year period.

To assess the pattern of attrition and possible implication for the results, we compared the baseline characteristics of those lost to follow-up and not lost to follow-up (Table 2 and section 3.2). The results showed that there was no systematic difference in most of the variables except bp control. Subsequently, all available data were included in the analysis without discarding partial records.

Predictors of stroke outcomes were examined using extensions of generalized linear models, such as the generalized estimating equation (GEE) model for functional dependency and cognitive function, and the linear mixed model for caregiver's burden. GEE was used because of its strength in handling longitudinal data in which study participants had varied follow-up. Also, parametric survival model of the Weibull distribution was used to examine predictors of mortality among the stroke survivors. The Weibull model was employed in this study because it assumes a baseline hazard function that increase or decrease monotonically. It was used for mortality which increases with age among stroke survivors. At the end of the study period, patients who had not experienced the event of interest (i.e. death) were right censored. Also, subjects who were lost to follow-up were censored as at the last time they were known to be alive. All inferential analyses were conducted at 5% significant level. For the multivariable model of each study outcome, significant factors from their respective univariable models were included. Specifically for mortality, occupation and ethnicity were controlled for. Regarding functional dependency, age of participant, history of previous stroke, cognitive function, and depression were adjusted for. The adjusted model for cognitive function controlled for age of participant, sex, education, marital status, occupation, income, stroke severity, history of previous stroke, and caregiver burden. Finally for caregiver burden, age, level of education, stroke severity, history of previous stroke, depression and cognitive function were included in the multivariable model. In addition, significant variables (*p* <0.05) from the univariable models were selected for multivariable models.

### 2.4 Ethical consideration

The health research and ethics committees at the study institution granted approval for the study (Federal Medical Center Abeokuta and University College Hospital, Ibadan) while written informed consent was obtained from each participant (Akinyemi et al., 2014).

## 3 Results

A total of 253 stroke survivors (FMC = 151; 59.7% and UCH = 102; 40.3%) were enrolled into the study during the 4-years period. Loss to follow-up which is a common challenge in longitudinal studies was documented and presented in Appendix A. The lost to follow-up rates varied by time-point after accounting for death. It was 19.8% after the baseline enrolment, 6.4%, 12.1%, 45.9% and 10% at 1^st^, 2^nd^, 3^rd^, and 4^th^ year follow-up period respectively (see Appendix A).

### 3.1 Baseline characteristics of all stroke survivors

Of the 253 participants in the study (see Table 1), the overall mean age was 60.20 (±9.8) years. Majority (70.0%) were between the ages of 50 and 69, male (62.6%), and domiciled in an urban area (96.8%). Also, most of the participants (90.5%) are of the Yoruba ethnic group. The clinical characteristics of the stroke survivors revealed that 56.5% of them had an uncontrolled blood pressure, and 13.2% of the participants had a prior history of stroke.

**Table 1:** Baseline background characteristics of all stroke survivors (*n* = 253).

**Socio-demographic profile** ***n*** **(%)**	
Mean age (SD)	60.20 (9.8)
**Age group (years)**	
<50	46 (18.3)
50–59	76 (30.3)
60–69	77 (30.7)
> = 70	52 (20.7)
**Site**	
Abeokuta	151 (59.7)
Ibadan	102 (40.3)
**Gender**	
Male	157 (62.6)
Female	94 (37.4)
**Education**	
No formal	26 (10.4)
Primary	60 (24.1)
Secondary	69 (27.7)
Tertiary	94 (37.8)
**Occupation**	
Not working	51 (21.4)
Artisan	26 (10.9)
Professional/civil servant	60 (25.2)
Trading/unskilled	76 (31.9)
Others	25 (10.5)
**Domicile**	
Rural	8 (3.2)
Urban	241 (96.8)
**Marital status**	
Married	218 (86.5)
Separated/Divorced	5 (2.0)
Widow/Widower	29 (11.5)
**Ethnicity**	
Yoruba	229 (90.5)
Non-Yoruba	24 (9.5)
**Monthly income**	
<= 10,000	51 (21.1)
10,001–25,000	62 (25.6)
25,001–50,000	71 (29.3)
50,001–100,000	30 (12.4)
100,001–150,000	14 (5.8)
> = 150,000	14 (5.8)
Lives alone	17 (6.77)
Lives with spouse/spouse and children	204 (81.27)
Lives with extended family	30 (11.95)
**Clinical profile** ***n*** **(%)**	
**Previous stroke**	
No	184 (86.8)
Yes	28 (13.2)
**Stroke type** ***n*** = **149**	
Ischemic	124 (83.2)
Hemorrhagic	25 (16.8)
**Obesity (bmi**>**30 kg/m**^2^**)**	
No	188 (77.4)
Yes	55 (22.6)
**Stroke severity**	
Severe	3 (1.4)
Moderate	46 (21.2)
Mild	168 (77.4)
**Alcoholism**	
No	112 (47.9)
Yes	122 (52.1)
**Smoking**	
No	183 (81.0)
Yes	43 (19.0)
**Hypertension**	
Systolic BP Mean (SD)	148 (32.5)
Diastolic BP Mean (SD)	88.26 (15.9)
**Uncontrolled blood pressure (SBP** ≥**140; DBP** ≥**90)**	
Normal	110 (43.5)
Hypertensive	143 (56.5)
**Cognitive function**	
Normal	125 (49.8)
Impaired	126 (50.2)
**Domain-specific cognitive impairment**	
Executive	45 (17.8)
Language	36 (14.2)
Memory	81 (32.0)
Visuospatial	92 (36.5)
**Depression**	
Not depressed	148 (60.7)
Depressed	96 (39.3)

### 3.2 Comparison of baseline characteristics of study participants

We investigated if there was systematic difference in the baseline characteristics of participants lost to follow-up and those who were not lost to follow-up at the end of the fourth year. As shown in Table 2, there is no significant difference (*p* > 0.05) in nearly all the baseline characteristics of those who were followed till the end of the study or their experience of death and those who were lost to follow-up.

**Table 2:** Comparison of study participants who completed the study and lost-to follow-up at end of study.

**Characteristics**	**Survivors who completed the study *n =* 138 *n* (%)**	**Survivors who were lost to follow up *n =* 115 *n* (%)**	**χ^2^**	***p-*value**
Average age (years)	59.5 ± 9.8	60.9 ± 9.7		0.288
**Center**				
Abeokuta	101 (73.2)	50 (43.5)	23.01	<0.001
Ibadan	37 (26.8)	65 (56.5)		
**Gender**				
Male	85 (62.5)	72 (62.6)	0.001	0.986
Female	51 (37.5)	43 (37.4)		
**Education**				
No formal	9 (6.7)	17 (14.8)	4.34	0.227
Primary	33 (24.6)	27 (23.5)		
Secondary	39 (29.1)	30 (26.1)		
Tertiary	53 (39.6)	41 (35.6)		
**Occupation**				
Not working	27 (21.3)	24 (21.6)	5.19	0.268
Artisan	14 (11.0)	12 (10.8)		
Professional/civil servant	33 (56.0)	27 (24.3)		
Trading/unskilled	35 (27.6)	41 (36.9)		
Others	18 (14.2)	7 (6.3)		
**Domicile**				
Rural	5 (3.7)	3 (2.7)	0.20	0.649
Urban	131 (96.3)	110 (97.3)		
**Marital status**				
Married	125 (91.2)	93 (80.9)	6.31	0.043
Separated/Divorced	1 (0.7)	4 (3.5)		
Widow/Widower	11 (8.0)	18 (15.6)		
**Ethnicity**				
Yoruba	127 (92.0)	102 (88.7)	0.81	0.368
Non-Yoruba	11 (8.0)	13 (11.3)		
**Monthly income**				
≤ 10,000	21 (16.0)	30 (27.0)	5.97	0.308
10,000–25,000	37 (28.2)	25 (22.5)		
25,001–50,000	39 (28.8)	32 (28.8)		
50,001–100,000	16 (12.2)	14 (12.6)		
100,001–150,000	10 (7.6)	4 (3.6)		
≥150,000	8 (6.1)	6 (5.4)		
**Living arrangement**				
Lives alone	7 (5.1)	10 (8.7)	2.23	0.327
Lives with spouse/spouse and children	115 (84.6)	89 (77.4)		
Lives with extended family	14 (10.3)	16 (13.9)		
**Previous stroke**				
No	101 (89.4)	83 (83.8)	1.41	0.234
Yes	12 (10.6)	16 (16.2)		
**Stroke subtype**				
Ischemic	64 (83.1)	60 (83.3)	0.01	0.972
Hemorrhagic	13 (16.9)	12 (16.7)		
**Stroke severity**				
Severe	2 (1.7)	1 (1.0)	2.09	0.350
Moderate	21 (17.6)	25 (25.5)		
Mild	96 (80.7)	72 (73.5)		
**Obesity**				
No	98 (75.4)	90 (79.6)	0.62	0.428
Yes	32 (24.6)	23 (20.4)		
**Alcohol**				
No	69 (53.5)	43 (40.9)	3.64	0.056
Yes	60 (46.5)	62 (59.1)		
**Smoking**				
No	102 (84.3)	81 (77.1)	1.86	0.172
Yes	19 (15.7)	24 (22.9)		
**Uncontrolled bp**				
Normal	70 (50.7)	40 (34.8)	6.48	0.011
Hypertensive	68 (49.3)	75 (65.2)		

### 3.3 Four-year trajectory of key stroke outcomes among survivors

Among all the stroke survivors, mortality was 8.30% (i.e 21 out of the 253 patients at baseline), 15/186 (8.06%) at 1 year follow-up, 36/160 (22.50%), 24/109 (22.02%), and 6/46 (13.04%) at 3, 3, and 4-years post-stroke respectively (see Table 3). The proportion of stroke survivors who were cognitively impaired decreased over time. It was 126/251 (50.2%) at baseline, 69/160 (43.1%), 45/109 (41.3%), 18/46 (39.1%), 12/36 (33.3%) at first, second, third and fourth-year follow-up respectively.

**Table 3:** Four-year trajectory of major stroke outcomes among survivors.

**Study outcomes**	**Follow up time**					
	**Baseline (*****n** =* **253)**	**6 month (*****n** =* **186)**	**1**^st^ **year (*****n** =* **160)**	**2**^nd^ **year (*****n** =* **109)**	**3**^rd^ **year (*****n** =* **46)**	**4**^th^ **year (*****n** =* **36)**
Mortality *n* (%)		21/253 (8.30)	15/186 (8.06)	36 (160 (22.50)	24/109 (22.02)	6/46 (13.04)
Cumulative mortality probability (95% C.I)		13.8 (10.08–18.63)	24.8 (19.93–30.57)	31.9 (26.53–37.98)	39.4 (33.7–45.7)	45.3 (39.42–51.64)
**Caregiver burden** ***n*** **(%)**						
Low level stress	97 (46.4)	67 (77.9)	88 (80.0)	56 (86.2)	28 (93.3)	22 (88.0)
High level stress	112 (53.6)	19 (22.1)	22 (20.0)	9 (13.9)	2 (6.7)	3 (12.0)
**Functional dependency (BI)** ***n*** **(%)**						
Independent	157 (76.6)	133 (87.5)	117 (86.7)	77 (84.6)	28 (77.8)	23 (76.7)
Dependent	48 (23.4)	19 (12.5)	18 (13.3)	14 (15.4)	8 (22.2)	7 (23.3)
**Stroke severity** ***n*** **(%)**						
Severe	3 (1.4)	1 (0.6)	2 (1.4)	2 (2.2)	0	0
Moderate	46 (21.2)	22 (13.2)	27 (19.2)	19 (20.4)	11 (31.4)	11 (36.7)
Mild	168 (77.4)	144 (86.2)	112 (79.4)	72 (77.4)	24 (68.6)	19 (63.3)
**Cognitive function** ***n*** **(%)**						
Normal	125 (49.8)	99 (53.2)	91 (56.9)	64 (58.7)	28 (60.9)	24 (66.7)
Impaired	126 (50.2)	87 (46.8)	69 (43.1)	45 (41.3)	18 (39.1)	12 (33.3)
**Depression** ***n*** **(%)**						
Not depressed	148 (60.7)	113 (61.1)	103 (64.8)	69 (64.5)	33 (73.3)	23 (63.9)
Depressed	96 (39.3)	72 (38.9)	56 (35.2)	38 (35.5)	12 (26.7)	13 (36.1)

### 3.4 Multivariable analysis of factors associated with stroke outcomes

#### 3.4.1 Mortality

Among all the stroke survivors, the cumulative probability of experiencing mortality was 13.8% (95% C.I = 10.08–18.63) (see Table 3) at 9-months post-stroke (i.e first 6-month follow up), 24.8% (95% CI = 19.93–30.6), 31.9% (95%CI = 26.53–37.98), 39.4% (95% CI = 3.65–45.7) and 45.3% (95% CI = 39.42–51.6) at 1, 2, 3, and 4-years post-stroke respectively. While majority (more than 50%) of the stroke survivors lived more than 4 years after the ictus (Appendix B1), those of them who were cognitively impaired experienced mortality earlier than their non-cognitively impaired counterparts (Appendix B2). Also, as seen in Table 4, results from the Weibull survival model revealed that after controlling for ethnicity, being an artisan as compared to a professional (aHR = 2.16; 95% CI = 1.21–3.84), increases the likelihood of death.

**Table 4:** Multivariable analysis of factors associated with mortality.

**Variables**	**Unadjusted HR (95% CI)**	***p-*value**	**Adjusted HR (95% CI)**	***p-*value**
Age (years)	1.02 (0.99–1.03)	0.065		
**Site**				
Abeokuta	ref			
Ibadan	0.76 (0.55–1.04)	0.094		
**Gender**				
Male	ref			
Female	1.02 (0.74–1.42)	0.879		
**Domicile**				
Rural	ref			
Urban	0.87 (0.38–1.96)	0.735		
**Education**				
No formal education	ref			
Primary	1.14 (0.66–1.97)	0.620		
Secondary	0.77 (0.44–1.34)	0.364		
Tertiary	0.77 (0.45–1.30)	0.340		
**Marital status**				
Married	ref			
Separated/Divorced	1.42 (0.53–3.86)	0.481		
Widow/Widower	1.20 (0.75–1.89)	0.447		
**Occupation**				
Not working	1.46 (0.88–2.42)	0.137	1.44 (0.83–2.38)	0.154
Professional	Ref		Ref	
Artisan	2.226 (1.27–4.02)	0.005	2.16 (1.21–3.84)	0.009
Trading/unskilled laborer	1.49 (0.94–2.37)	0.087	1.52 (0.95–2.41)	0.076
Others	1.35 (0.71–2.57)	0.355	1.31 (0.69–2.49)	0.405
**Ethnicity**				
Yoruba	ref		ref	
Non-Yoruba	1.94 (1.02–3.68)	0.043	1.93 (0.98–3.80)	0.057
**Monthly income**				
≤ 10,000	ref			
10,001–25,000	0.74 (0.46–1.18)	0.213		
25,001–50,000	0.71 (0.31–1.58)	0.401		
50,001–100,000	0.79 (0.51–1.24)	0.319		
100,000–150,000	0.68 (0.37–1.21)	0.195		
≥150,000	1.15 (0.58–2.26)	0.675		
**Living arrangement**				
Lives alone	ref			
Lives with spouse/spouse and children	0.60 (0.35–1.04)	0.071		
Lives with extended family	1.03 (0.54–1.95)	0.925		
**Stroke severity**				
Severe	0.51 (0.07–3.62)	0.498		
Moderate	0.82 (0.54–1.24)	0.355		
Mild	ref			
**Previous stroke**				
No	ref			
Yes	1.21 (0.74–1.96)	0.443		
**Obesity**				
Not	ref			
Yes	0.92 (0.62–1.36)	0.683		
**Alcoholism**				
No	ref			
Yes	0.84 (0.61–1.17)	0.311		
**Smoking**				
No	ref			
Yes	0.80 (0.51–1.25)	0.338		
**Cognitive status**				
Normal	ref			
Impaired	1.14 (0.83–1.56)	0.414		
**Functional dependency**				
Independent	ref			
Dependent	1.12 (0.74–1.71)	0.565		
**Depression**				
Not depressed	ref			
Depressed	0.94 (0.67–1.31)	0.726		

#### 3.4.2 Functional dependency

Results from the GEE model after controlling for cognitive function and depression among the participants showed that the higher the stroke survivors' age, the higher their likelihood of being functionally dependent (aOR = 1.04; 1.01–1.08). Meanwhile, history of a previous stroke (aOR = 2.24, 95% CI = 1.22–4.13) also increased the likelihood of functional dependency in the cohort. These results are presented in Table 5.

**Table 5:** Factors associated with functional dependency among stroke survivors.

**Characteristics**	**Unadjusted odds ratio**	***p-*value**	**Adjusted odds ratio**	***p-*value**
Age (years)	1.06 (1.03–1.09)	<0.001	1.04 (1.01–1.08)	0.010
**Site**				
Abeokuta	ref			
Ibadan	1.44 (0.78–2.64)	0.243		
**Gender**				
Male	ref			
Female	1.07 (0.57–2.03)	0.824		
**Domicile**				
Rural	ref			
Urban	0.38 (0.09–1.47)	0.160		
**Education**				
No formal education	ref			
Primary	0.79 (0.30–2.07)	0.627		
Secondary	0.37 (0.13–1.04)	0.059		
Tertiary	0.44 (0.17–1.15)	0.093		
**Marital status**				
Married	ref			
Seperated/Divorced	0.36 (0.02–8.36)	0.523		
Widow/Widower	1.51 (0.63–3.58)	0.352		
**Occupation**				
Not working	1.15 (0.45–2.94)	0.776		
Professional	ref			
Artisan	1.01 (0.31–3.26)	0.941		
Trading/unskilled laborer	1.21 (0.51–2.83)	0.579		
Others	0.88 (0.26–3.01)	0.786		
**Ethnicity**				
Yoruba	1.34 (0.42–4.29)	0.621		
Non-Yoruba	ref			
**Monthly income**				
≤ 10,000	ref			
10,001–25,000	1.05 (0.41–2.67)	0.915		
25,001–50,000	1.08 (0.44–2.68)	0.864		
50,001–100,000	0.81 (0.25–2.65)	0.725		
100,000–150,000	1.55 (0.39–6.22)	0.533		
≥150,000	1.21 (0.29–4.92)	0.792		
**Living arrangement**				
Lives alone	ref			
Lives with spouse/spouse and children	0.50 (0.16–1.52)	0.221		
Lives with extended family	0.75 (0.19–2.81)	0.668		
**Stroke severity**				
Severe	ref			
Moderate	1.92 (0.45–8.12)	0.376		
Mild	0.29 (0.07–1.24)	0.096		
**Previous stroke**				
No	ref		ref	
Yes	2.36 (1.34–4.14)^*^	0.003	2.24 (1.22–4.13)	0.010
**Obesity**				
Not obese	ref			
Obese	0.81 (0.51–1.30)	0.386		
**Alcohol**				
No	ref			
Yes	0.51 (0.26–1.01)	0.51		
**Smoking**				
No	ref			
Yes	0.66 (0.26–1.72)	0.400		
**Cognitive function**				
Normal	ref		ref	
Impaired	1.77 (1.26–2.49)^*^	0.001	1.47 (0.94–2.29)	0.086
**Depression**				
Not depressed	ref		ref	
Depressed	1.37 (1.01–1.84)	0.041^*^	1.28 (0.89–1.85)	0.173

^*^Symbol denotes statistical significance at 5% significance level.

#### 3.4.3 Cognitive impairment

After controlling for age, sex, marital status, income, and stroke severity, the result of the generalized estimating equation model showed that there was a dose-response, negative relationship between education and cognitive impairment (i.e., the higher the educational attainment, the lower the chance of being cognitively impaired). It was observed that survivors who were not working were 3.7 times more likely than professionals to be cognitively impaired (aOR = 3.70, 95% CI = 1.22–11.21). Meanwhile, stroke survivors with repeat stroke (aOR = 1.92; 95% CI = 1.03–3.57) and those with higher caregiver burden (aOR = 1.92 95% CI = 1.03–3.57) were more likely to be cognitively impaired (see Table 6).

**Table 6:** Factors associated with cognitive function among stroke survivors.

**Characteristics**	**Unadjusted odds ratio**	***p-*value**	**Adjusted odds ratio**	***p-*value**
Age (years)	1.06 (1.03–1.08)	<0.001	0.98 (0.94–1.03)	0.589
**Site**				
Abeokuta	ref			
Ibadan	1.09 (0.72–1.67)	0.676		
**Gender**				
Male	ref		ref	
Female	1.71 (1.11–2.64)^*^	0.015	1.41 (0.66–3.01)	0.369
**Domicile**				
Rural	ref			
Urban	1.78 (0.52–6.13)	0.357		
**Education**				
No formal education	ref		ref	
Primary	0.13 (0.04–0.41)^*^	0.001	0.04 (0.01–0.38)	0.004
Secondary	0.08 (0.03–0.25)^*^	<0.01	0.01 (0.01–0.09)	<0.001
Tertiary	0.05 (0.02–0.16)^*^	<0.01	0.01 (0.01–0.03)	<0.001
**Marital status**				
Married	ref		ref	
Separated/divorced	1.92 (0.44–8.46)	0.387	2.76 (0.29–26.16)	0.376
Widow/widower	2.13 (1.08–4.18)^*^	0.028^*^	0.24 (0.06–0.95)	0.043
**Occupation**				
Not working	1.62 (0.86–3.06)	0.134	1.91 (0.70–5.21)	0.205
Professional	ref		ref	
Artisan	0.69 (0.30–1.55)	0.369	0.10 (0.02–0.42)	0.002
Trading/unskilled laborer	2.02 (1.13–3.59)^*^	0.017	0.19 (0.06–0.60)	0.004
Others	1.23 (0.55–2.73)	0.612	0.62 (0.18–2.05)	0.437
**Ethnicity**				
Yoruba	0.68 (0.33–1.40)	0.293		
Non-Yoruba	ref			
**Monthly income**				
≤ 10,000	ref		ref	
10,001–25,000	0.55 (0.29–1.04)	0.066	0.50 (0.16–1.48)	0.212
25,001–50,000	0.32 (0.17–0.60)^*^	<0.001	1.67 (0.33–8.33)	0.527
50,001–100,000	0.41 (0.19–0.88)^*^	0.022	0.81 (0.28–2.34)	0.704
100,001–150,000	0.52 (0.19–1.43)	0.203	0.67 (0.19–2.33)	0.532
≥150,000	0.21 (0.07–0.61)^*^	0.004	0.52 (0.10–2.58)	0.424
**Living arrangement**				
Lives alone	ref			
Lives with spouse/spouse and children	0.89 (0.37–2.13)	0.797		
Lives with extended family	2.33 (0.81–6.70)	0.118		
**Stroke severity**				
Severe	1.09 (0.38–3.14)	0.877	1.59 (0.41–6.11)	0.495
Moderate	1.51 (1.07–2.13)^*^	0.018	1.16 (0.75–1.80)	0.496
Mild	ref		ref	
**Previous stroke**				
No	ref		ref	
Yes	2.63 (1.52–4.56)^*^	0.001	2.23 (1.12–4.45)	0.022
**Obesity**				
Not obese	ref			
Obese	1.42 (0.97–2.08)	0.072		
Caregiver burden	1.02 (1.01–1.02)^*^	<0.001	1.02 (1.01–1.03)	<0.001
**Depression**				
Not depressed	ref			
Depressed	1.14 (0.91–1.44)	0.259		

^*^Symbol denotes statistical significance at 5% significance level.

#### 3.4.4 Caregiver burden

Socio-demographic, clinical and lifestyle factors (see Table 7) that were significant in the univariable model were controlled for in the multivariable model. These controlled factors include, level of education, and stroke severity. According to results from the linear mixed model, older age (β = 9.53; 95% CI = 1.28–17.78), history of previous stroke (β = 22.12; 95%CI = 13.68–30.57), depression (β = 6.77; 95%CI = 2.29–11.25) and cognitive impairment (β = 10.89; 95% CI = 5.99–15.80) are significantly associated with increased caregiver burden.

**Table 7:** Factors associated with caregiver burden.

**Characteristics**	**Model I (univariable)**	***p-*value**	**Model II**	***p-*value**	**Model III**	***p-*value**
Age (years)	0.61 (0.32–0.91)	**<0.001**	0.35 (0.08, 0.62)	0.011	0.33 (0.05–0.60)	0.017
**Site**						
Abeokuta	ref					
Ibadan	2.48 (−3.25–8.21)	0.396				
**Gender**						
Male	ref					
Female	−1.62 (−7.53–4.27)	0.588				
**Domicile**						
Rural	ref					
Urban	−6.72 (−23.09–9.66)	0.421				
**Education**						
No formal education	ref		ref		ref	
Primary	−1.20 (−11.49–9.08)	0.819	4.05 (−4.78, 12.89)	0.369	4.20 (−4.51–12.91)	0.345
Secondary	−8.61 (−18.71–1.48)	0.094	1.42 (−7.40, 10.24)	0.752	1.36 (−7.35–10.07)	0.759
Tertiary	−11.95 (−21.68; −2.23)^*^	0.016	0.09 (−8.78, 8.98)	0.983	0.17 (−8.70–9.04)	0.970
**Marital status**						
Married	ref					
Separated/Divorced	−3.01 (−21.42–15.41)	0.749				
Widow/Widower	7.53 (−1.33–16.39)	0.096				
**Occupation**						
Not working	5.22 (−3.33–13.78)	0.231				
Professional	ref					
Artisan	4.61 (−6.19–15.41)	0.403				
Trading/unskilled laborer	5.66 (−2.11–13.43)	0.153				
Others	1.97 (−8.76–12.69)	0.719				
**Ethnicity**						
Yoruba	−3.41 (−12.89–6.08)	0.481				
Non-Yoruba	ref					
**Monthly income**						
≤ 10,000	ref					
10,001–25,000	−1.33 (−9.99–7.32)	0.763				
25,001–50,000	−1.36 (−9.74–7.02)	0.750				
50,001–100,000	−8.81 (−18.85–1.23)	0.086				
100,001–150,000	−4.87 (−18.83–9.09)	0.494				
≥150,000	−7.17 (−21.03–6.69)	0.311				
**Living arrangement**						
Lives alone	ref					
Lives with spouse/spouse and children	−2.99 (−15.95–9.98)	0.651				
Lives with extended family	−2.43 (−17.26–12.39)	0.748				
**Stroke severity**						
Severe	19.95 (−0.08–39.99)	0.051	14.35 (−4.55, 33.26)	0.137	14.94 (−4.08–33.97)	0.124
Moderate	17.25 (11.80–22.69)^*^	<0.01	15.98 (10.64, 21.32)	<0.001	15.86 (10.55–21.17)	<0.001
Mild	ref		ref		ref	
**Previous stroke**						
No	ref		ref		ref	
Yes	29.28 (20.49–38.08)^*^	<0.001	21.47 (13.05, 29.90)	<0.001	22.03 (13.66–30.41)	<0.001
**Obesity**						
Not obese	ref					
Obese	−2.17 (−8.54–4.19)	0.503				
**Depression**						
Not depressed	ref		ref		ref	
Depressed	8.54 (4.11–12.97)^*^	<0.001	6.45 (2.00, 10.91)	0.004	6.18 (1.73–10.64)	0.006
**Cognitive function**						
Normal	ref		ref			
Impaired	15.79 (11.14–20.45)^*^	<0.001	10.89 (6.02, 15.76)	<0.001		
**Cognitive impairment (executive)**						
No	ref				ref	
Yes	13.92 (7.62–20.22)^*^	<0.001			2.91 (−3.84–9.67)	0.398
**Cognitive impairment (language)**						
No	ref					
Yes	12.57 (5.59–19.55)^*^	<0.001			ref	
Cognitive impairment (memory)					−1.77 (−9.46–5.92)	0.652
No	ref				ref	
Yes	13.89 (8.94–18.85)^*^	<0.001			4.34 (−1.74–10.42)	0.162
**Cognitive impairment (visuospatial)**						
No	ref				ref	
Yes	14.17 (9.23–19.11)^*^	<0.001			8.64 (2.81–14.46)	0.004

^*^Symbol denotes statistical significance at 5% significance level.

## 4 Discussion

Stroke requires long-term management (GBD 2019 Stroke Collaborators, 2021; Kim et al., 2020; Hill et al., 2022); however, estimates of different outcomes after stroke in the long term, beyond 1 year, are lacking, with most of the existing data on stroke outcomes being limited to short-term cohort studies (usually up to 1 year), as well as focusing on disability alone or only a few outcome measures. This study thus, fill the gap by providing longitudinal data on health outcomes up to 4 years among stroke survivors.

Findings from this 4-year longitudinal study of stroke survivors in Nigeria present the trajectory of health outcomes and predictors of long-term outcomes among older adult stroke survivors. Most of the stroke survivors were males, had ischemic stroke and were hypertensive. These are not uncommon patterns among stroke patients (Barker-Collo et al., 2015; Sarfo et al., 2018). In a previous cross-sectional study, nearly 33% of the Nigerian stroke survivors had post stroke depression (Oni et al., 2018), similar to the proportion of adult stroke survivors who had depression in our study from baseline up to 2 years follow-up.

Our result showed that the peak proportion of adult stroke survivors who died was at second (22.5%) and third year (22.0%) follow-up respectively. Meanwhile, the cumulative probability of death among the survivors markedly increased to 45.3% at the end of the study, highlighting the persistent vulnerability of stroke survivors beyond the acute phase. This suggests that mid- to long-term interventions are essential to improving survival in this population. One key factor for higher likelihood of mortality was being an artisan. This association might be a reflection of the usually experienced occupational and socioeconomic vulnerabilities among artisans. For instance, most artisans are often with little or no formal education. Furthermore, artisanal work might entail extended physical labor under dangerous circumstances including dust exposure, chemical exposure, lifting of heavy objects, or risk of injury, especially in locations lacking strong occupational safety regulations. These cumulative exposures and constrained access to preventive or curative health services could reasonably raise death risks.

Another key finding from our study was that the proportion of survivors who were functionally dependent increased among the stroke survivors during the 4 year period. Age, previous history of stroke, cognitive impairment and depressive symptom independently predicted functional dependency among stroke survivors. This corroborates findings of previous studies from Nigeria and elsewhere that older age (Ojule and Daniel-Amadi, 2022; Li et al., 2016) and a history of previous stroke (Ojule and Daniel-Amadi) are related to being functionally dependent among stroke survivors. Another similar study from Nigeria Ojagbemi and Owolabi, (2013), found that cognitive impairment and depressive symptom are related to functional dependency among stroke survivors. Meanwhile, even after controlling for other factors, older age and history of previous stroke remained significant predictors of functional dependency. This highlights that advanced age of the survivor and a prior experience of stroke markedly impact on the functional stability of a stroke survivor. Although the rise in the proportion of functionally dependent stroke survivors among study participants is in sharp contrasts with the results of Sennfält et al. (2019). Several factors could explain these discrepancies. Variations in the demographic and clinical characteristics of study populations, such as age, comorbidities, and stroke severity, can influence outcomes. Other possible reasons could be due to increase in the number of stroke units and secondary prevention (such as anti-coagulant therapy) in the Swedish population. Post-stroke depression (PSD) was significantly associated with poorer functional independence in activities of daily living (ADL). This pattern mirrors previous findings from Nigeria (Ezema et al., 2019). Depression after stroke commonly causes reduced motivation, which directly reduces a survivor's willingness and energy to participate in rehabilitation and to practice motor tasks that foster ADL recovery. Consequently, reduced practice/engagement slows motor learning and functional gains, producing higher dependency.

The proportion of our participants who were cognitively impaired appeared to reduce during the period. This should be taken with caution because of the substantial attrition in the cohort. Participants lost to follow-up were most likely to have had poorer outcomes. Low educational attainment and a history of previous stroke were significant predictors of cognitive performance among our participants, aligning with previous findings (Kalaria et al., 2016; Pendlebury and Rothwell, 2019; Qu et al., 2015). This implied that participants with little or no education had limited cognitive reserve and were thus at higher risk of being cognitively impaired following a stroke occurrence. Education strengthens the brain's ability to cope with age-related or disease-related pathology. In practical terms, two people with equal cognitive impairment pathology can show very different clinical performance because the more “reserve” one can maintain function longer despite the same pathology. This concept is well reviewed and widely cited (Godinho et al., 2022; Thow et al., 2017). Formal schooling trains and repeatedly engages executive, memory, language and reasoning systems across decades, encouraging network efficiency and inter-regional connectivity. Also, the risk of being cognitively impaired was higher among the survivors, who had history of a previous stroke. Our findings also revealed that caregiver burden and the female gender were associated with reduced cognitive performance among stroke survivors.

Poor stroke outcomes, including functional dependence, cognitive impairment, and depression were high at 3 months post-stroke. This probably explains why caregiver burden was highest at the same period. A stroke survivor who is cognitively impaired, functionally impaired and experiencing depression would likely burden the caregiver the more. This identified relationship highlights the critical period shortly after a stroke wherein both stroke survivors and their caregivers face the most significant challenges.

Consistent with the findings of McCullagh (McCullagh et al., 2005), older age is an independent predictor of caregiver burden as well as depression. Older individuals, regardless of their health condition, tend to have weaker immune systems, exacerbating the challenges for stroke survivors and subsequently increasing the stress on caregivers. Factors such as advanced age, history of a recurrent stroke, cognitive impairment, and depression independently predict functional impairment among stroke survivors, supporting the findings of other previous studies (Ojagbemi and Owolabi, 2013; Sennfält et al., 2019). Furthermore, patient's depression brings additional emotional burden to the caregiver. The caregiver will have to manage mood symptoms, agitation, social withdrawal, suicide risk or repeated help-seeking; all of which increases subjective burden beyond physical caregiving tasks. Patient's psychological symptoms have been identified as predictors of higher caregiver burden (Oni et al., 2019) in Nigeria. Notably, caregiver burden and patient depression can interact bidirectionally: a depressed, dependent patient increases caregiver's strain while caregiver stress (and poor support) can worsen patient mood and recovery. This kind of reciprocity has been described in stroke caregiving research (Fang et al., 2022).

Finally, for most of the outcomes that were examined in this study, older age of the stroke survivor and a history of a previous stroke independently predicted poorer outcomes among stroke survivors. In contrast, having higher educational level was associated with better outcomes among stroke survivors.

## 5 Limitation and strength of the study

One major limitation of this study is that which is common to longitudinal studies—attrition. While the authors endeavored to account for each participant that was recruited into the study, a significant number was lost to follow up especially during the third year. Those who were lost to follow-up were patients who either relocated to a different place for further treatment or rehabilitation and at such stopped visiting the study centers or those who could not be reached through their means of contact or patients who possibly had died as a result of complications from stroke or natural cause. Another potential reason for the high attrition rate could be the common spiritual belief that a neurological disorder as stroke is a spiritual attack. Patients and caregivers may discontinue with orthodox treatment after some time while the patient is yet to fully recover.

Although data on history of previous stroke was collected at baseline further history of recurrences could not be ascertained as a result of losses to follow up. Hence, we are unable to extensively quantify stroke reccurrence among the participants. This subsequently limits the extent of generalizability of the relationship between recurrent stroke and the study outcomes. Despite these limitations, the study filled the gap in paucity of longitudinal data on stroke outcomes from the African region where such research is challenging to undertake given limitations in infrastructure and health care utilization. Additionally, the study investigated multiple health outcomes among the patients, compared to most studies that focus commonly on one outcome—mortality and/functional dependency.

## 6 Conclusion

The results suggest a definite demand for mid- and long-term interventions extending stroke management outside of hospital discharge. Multidisciplinary teams could help stroke units strengthen secondary prevention strategies (Adeniji et al., 2023). The study identified that individuals' educational attainment was important to improve stroke outcomes, as this has been shown to create cognitive reserve in stroke survivors in a dose-response manner. Higher educational attainment reduces the clinical risk of cognitive impairment primarily by building cognitive reserve as well as delays the onset of detectable impairment (Godinho et al., 2022; Thow et al., 2017). Consequently, education is highly correlated with occupational complexity. For instance, more educated people tend to hold cognitively demanding jobs and engage more in leisure activities that maintain cognition across the lifespan. Since education and cognitive reserve have a major impact on results, public health and education policies that raise stroke awareness and health literacy in populations, particularly among socioeconomically disadvantaged groups like artisans may be strengthened. Additionally, secondary stroke prevention is crucial since stroke recurrence is a strong predictor of major stroke outcomes (functional dependency, cognitive function, and caregiver burden). Finally, special consideration is needed for the treatment and care of older stroke survivors, knowing that their weak immune system could make them more vulnerable to poor stroke outcomes. Based on findings from this study, it is recommended that good educational attainment should be encouraged and made readily available to the populace to build their cognitive reserve.

Conclusively, although resource constraints continue to be a problem, incremental but coordinated actions such as expanding stroke units in teaching hospitals, integrating caregiver support into daily practice, and giving secondary prevention first priority in primary care are crucial actions that can significantly improve long-term results for stroke survivors in Nigeria.

## Data Availability

The raw data supporting the conclusions of this article will be made available by the authors, without undue reservation.

## References

[B1] Adeniji O. Adeleye O. Akinyemi J. Otubogun F. Ogunde G. Ogunrombi M. . (2023). Organized multi-disciplinary stroke team care improves acute stroke outcomes in resource limited settings; results of a retrospective study from a Nigerian tertiary hospital. J. Stroke Cerebrovasc. Dis. 32:107307. doi: 10.1016/j.jstrokecerebrovasdis.2023.107307 37633206

[B2] Akinyemi R. O. Allan L. Owolabi M. O. Akinyemi J. O. Ogbole G. Ajani A. . (2014). Profile and determinants of vascular cognitive impairment in African stroke survivors: the CogFAST Nigeria study. J. Neurol. Sci. 346, 1–2. doi: 10.1016/j.jns.2014.08.042 25238666

[B3] Akinyemi R. O. Ovbiagele B. Adeniji O. A. Sarfo F. S. Abd-Allah F. Adoukonou T. . (2021). Stroke in Africa: profile, progress, prospects and priorities. Nat. Rev. Neurol. 17, 634–656. doi: 10.1038/s41582-021-00542-4 34526674 PMC8441961

[B4] American Psychiatric Association (1994). Diagnostic and Statistical Manual of Mental Disorders, 4th Edn. Washington, DC.

[B5] Barker-Collo S. Bennett D. A. Krishnamurthi R. V. Parmar P. Feigin V. L. Naghavi M. . (2015). Sex differences in stroke incidence, prevalence, mortality and disability-adjusted life years: results from the Global Burden of Disease Study 2013. Neuroepidemiology 45, 203–214. doi: 10.1159/000441103 26505984 PMC4632242

[B6] BeLue R. Okoror T. A. Iwelunmor J. Taylor K. D. Degboe A. N. Agyemang C. Ogedegbe G. (2009). An overview of cardiovascular risk factor burden in Sub-Saharan African countries: a socio-cultural perspective. Glob. Health 5:10. doi: 10.1186/1744-8603-5-10 19772644 PMC2759909

[B7] Donkor E. S. (2018). Stroke in the 21st century: a snapshot of the burden, epidemiology, and quality of life. Stroke Res. Treat. 22018:3238165. doi: 10.1155/2018/3238165 30598741 PMC6288566

[B8] Ezema C. I. Akusoba P. C. Nweke M. C. Uchewoke C. U. Agono J. Usoro G. (2019). Influence of post-stroke depression on functional independence in activities of daily living. Ethiop. J. Health Sci. 29, 841–846. doi: 10.4314/ejhs.v29i1.5 30700951 PMC6341441

[B9] Fang L. Dong M. Fang W. Zheng J. (2022). Relationships between care burden, resilience, and depressive symptoms among the main family caregivers of stroke patients: a cross-sectional study. Front. Psychiatry 13:960830. doi: 10.3389/fpsyt.2022.960830 36203823 PMC9530984

[B10] GBD 2019 Stroke Collaborators (2021). Global, regional, and national burden of stroke and its risk factors, 1990-2019: a systematic analysis for the Global Burden of Disease Study 2019. Lancet Neurol. 20, 795–820. doi: 10.1016/S1474-4422(21)00252-0 34487721 PMC8443449

[B11] Godinho F. Maruta C. Borbinha C. Pavão Martins I. (2022). Effect of education on cognitive performance in patients with mild cognitive impairment. Appl. Neuropsychol. Adult 29, 1440–1449. doi: 10.1080/23279095.2021.1887191 33721504

[B12] Gorelick P. B. Scuteri A. Black S. E. Decarli C. Greenberg S. M. Iadecola C. . (2011). Vascular contributions to cognitive impairment and dementia: a statement for healthcare professionals from the American Heart Association/American Stroke Association. Stroke 42, 2672–2713. doi: 10.1161/STR.0b013e3182299496 21778438 PMC3778669

[B13] Hachinski V. Iadecola C. Petersen R. C. Breteler M. M. Nyenhuis D. L. Black S. E. . (2006). National Institute of Neurological Disorders and Stroke-Canadian Stroke Network vascular cognitive impairment harmonization standards. Stroke 37, 2220–2241. doi: 10.1161/01.STR.0000237236.88823.47 16917086

[B14] Hill G. Regan S. Francis R. Mead G. Thomas S. Salman R. A. . (2022). Research priorities to improve stroke outcomes. Lancet Neurol. 21, 312–313. doi: 10.1016/S1474-4422(22)00044-8 35305334 PMC8926410

[B15] Kalaria R. N. Akinyemi R. Ihara M. (2016). Stroke injury, cognitive impairment and vascular dementia. Biochim. Biophys. Acta Mol. Basis Dis. 1862, 915–924. doi: 10.1016/j.bbadis.2016.01.015 26806700 PMC4827373

[B16] Kim S. E. Lee H. Kim J. Y. Lee K. J. Kang J. Kim B. J. . (2020). Three-month modified Rankin Scale as a determinant of 5-year cumulative costs after ischemic stroke: an analysis of 11,136 patients in Korea. Neurology 94:e978–e91. doi: 10.1212/WNL.0000000000011295 32029544

[B17] Li P. Zang X.-Y. Wang Y. Chai Q.-W. Wang J.-Y. Sun C.-Y. Zhang Q. (2016). Factors associated with activities of daily living among the disabled elders with stroke. Int. J. Nurs. Sci. 3, 29–34. doi: 10.1016/j.ijnss.2016.01.002

[B18] Lo J. W. Crawford J. D. Desmond D. W. Bae H. J. Lim J. S. Godefroy O. . (2022). Long-term cognitive decline after stroke: an individual participant data meta-analysis. Stroke 53, 1318–1327. doi: 10.1161/STROKEAHA.121.035796 34775838

[B19] McCullagh E. Brigstocke G. Donaldson N. Kalra L. (2005). Determinants of caregiving burden and quality of life in caregivers of stroke patients. Stroke 36, 2161–2166. doi: 10.1161/01.STR.0000181755.23914.53 16151029

[B20] Obembe A. Oluwatosin F. (2012). Reliability and concurrent validity of Modified Barthel Index in a Nigerian language. J. Niger. Soc. Physiother. 20, 21–24.

[B21] Ogunlana M. O. Dada O. O. Oyewo O. S. Odole A. C. Ogunsan M. O. (2014). Quality of life and burden of informal caregivers of stroke survivors. Hong Kong Physiother. J. 32, 6–12. doi: 10.1016/j.hkpj.2013.11.003

[B22] Ojagbemi A. Owolabi M. (2013). Predictors of functional dependency after stroke in Nigeria. J. Stroke Cerebrovasc. Dis. 22:e381–e387. doi: 10.1016/j.jstrokecerebrovasdis.2013.04.015 23680683

[B23] Ojule I. N. Daniel-Amadi U. (2022). Disability levels and associated factors of functional dependence among stroke survivors in South-South, Nigeria. Asian J. Adv. Res. Rep. 16, 12–21. doi: 10.9734/ajarr/2022/v16i330461

[B24] Okekunle A. P. Jones S. Adeniji O. Watkins C. Hackett M. Di Tanna G. L. . (2023). Stroke in Africa: a systematic review and meta-analysis of the incidence and case-fatality rates. Int. J. Stroke 18, 563–573. doi: 10.1177/17474930221147164 36503371 PMC10313746

[B25] Oni O. D. Olagunju A. T. Okpataku C. I. Erinfolami A. R. Adeyemi J. D. (2019). Predictors of caregiver burden after stroke in Nigeria: effect on psychosocial well-being. Indian J. Psychiatry 61, 457–464. doi: 10.4103/psychiatry.IndianJPsychiatry_395_18 31579140 PMC6767813

[B26] Oni O. D. Olagunju A. T. Olisah V. O. Aina O. F. Ojini F. I. (2018). Post-stroke depression: prevalence, associated factors and impact on quality of life among outpatients in a Nigerian hospital. S. Afr. J. Psychiatr. 24:1058. doi: 10.4102/sajpsychiatry.v24i0.1058 30263206 PMC6138133

[B27] Owolabi M. O. Akarolo-Anthony S. Akinyemi R. Arnett D. Gebregziabher M. Jenkins C. . (2015). The burden of stroke in Africa: a glance at the present and a glimpse into the future. Cardiovasc. J. Afr. 26, S27–S38. doi: 10.5830/CVJA-2015-038 25962945 PMC4557491

[B28] Owolabi M. O. Platz T. (2008). Proposing the Stroke Levity Scale: a valid, reliable, simple, and time-saving measure of stroke severity. Eur. J. Neurol. 15, 627–633. doi: 10.1111/j.1468-1331.2008.02140.x 18474078

[B29] Pendlebury S. T. Rothwell P. M. (2019). Incidence and prevalence of dementia associated with transient ischaemic attack and stroke: analysis of the population-based Oxford Vascular Study. Lancet Neurol. 18, 248–258. doi: 10.1016/S1474-4422(18)30442-3 30784556 PMC6390174

[B30] Qu Y. Zhuo L. Li N. Hu Y. Chen W. Zhou Y. . (2015). Prevalence of poststroke cognitive impairment in China a community-based, cross-sectional study. PLoS One 10:e0122864. doi: 10.1371/journal.pone.0122864 25874998 PMC4395303

[B31] Sarfo F. S. Ovbiagele B. Gebregziabher M. Wahab K. Akinyemi R. Akpalu A. . (2018). Stroke among young West Africans. Stroke 49, 1024–1027. doi: 10.1161/STROKEAHA.118.020783 29618553 PMC5916042

[B32] Sennfält S. Norrving B. Petersson J. Ullberg T. (2019). Long-term survival and function after stroke: a longitudinal observational study from the Swedish Stroke Register. Stroke 50, 62–69. doi: 10.1161/STROKEAHA.118.022913 30580719

[B33] Stolwyk R. J. Mihaljcic T. Wong D. K. Chapman J. E. Rogers J. M. (2021). Poststroke cognitive impairment negatively impacts activity and participation outcomes: a systematic review and meta-analysis. Stroke 52, 748–760. doi: 10.1161/STROKEAHA.120.032215 33493048

[B34] Sullivan M. T. (2002). Caregiver strain index (CSI). J. Gerontol. Nurs. 28, 4–5. doi: 10.3928/0098-9134-20020801-03 12349829

[B35] Thow M. E. Summers M. J. Saunders N. L. Summers J. J. Ritchie K. Vickers J. C. (2017). Further education improves cognitive reserve and triggers improvement in selective cognitive functions in older adults: the Tasmanian Healthy Brain Project. Alzheimers Dement 10, 22–30. doi: 10.1016/j.dadm.2017.08.004 29034310 PMC5633863

[B36] Uyttenboogaart M. Stewart R. E. Vroomen P. C. De Keyser J. Luijckx G. J. (2005). Optimizing cutoff scores for the Barthel index and the modified Rankin scale for defining outcome in acute stroke trials. Stroke 36, 1984–1987. doi: 10.1161/01.STR.0000177872.87960.61 16081854

[B37] Wang J. An Z. Li B. Yang L. Tu J. Gu H. . (2015). Increasing stroke incidence and prevalence of risk factors in a low-income Chinese population. Neurology 84, 374–381. doi: 10.1212/WNL.0000000000001175 25540314

[B38] Wilson J. T. Lindsay H.areendran, A. Grant M. Baird T. Schulz U. G. R. Muir K. W. Bone I. (2002). Improving the assessment of outcomes in stroke: use of a structured interview to assign grades on the modified Rankin Scale. Stroke 33, 2243–2246. doi: 10.1161/01.STR.0000027437.22450.BD 12215594

[B39] Wolfe C. D. Crichton S. L. Heuschmann P. U. McKevitt C. J. Toschke A. M. Grieve A. P. . (2011). Estimates of outcomes up to ten years after stroke: analysis from the prospective South London Stroke Register. PLoS Med. 8:e1001033. doi: 10.1371/journal.pmed.1001033 21610863 PMC3096613

[B40] World Stroke Organization (2024). Impact of Stroke. Available online at: https://www.world-stroke.org/world-stroke-day-campaign/about-stroke/impact-of-stroke#:~:text=Stroke%20is%20a%20leading%20cause,A%20stroke%20in%20their%20lifetime.andtext=Over%2012%20million%20people%20worldwide,Will%20die%20as%20a%20result

[B41] Yesavage J. A. Brink T. L. Rose T. L. Lum O. Huang V. Adey M. Leirer V. O. (1982). Development and validation of a geriatric depression screening scale: a preliminary report. J. Psychiatr. Res. 17, 37–49. doi: 10.1016/0022-3956(82)90033-4 7183759

