# Supplementary material for: Long term outcomes among African stroke survivors: 4 years follow up data from the CogFAST—Nigeria Study
Source: Front Stroke. 2025 Dec 19;4:1586814. doi: 10.3389/fstro.2025.1586814 (PMC12802744; doi:10.3389/fstro.2025.1586814)
Supplement: Supplementary file 1 [file Table_1.docx]

**Appendix A: Patients’ retention over the four-years study period**

Stroke survivors from UCH n=102 Stroke survivors from FMC n=151

Total number of stroke survivors who were enrolled into the study n=253

(died = 21 LTF = 46)

Number of stroke survivors 6 months after enrolment n=186

(died =15 LTF = 11)

Number of stroke survivors one year after enrolment n=160

(died = 36 LTF = 15)

Number of stroke survivors 2 years after enrolment n=109

(died =24 LTF = 39)

Number of stroke survivors 3 years after enrolment n=46

(died = 6 LTF = 4)

Number of str**oke patients 4 years after enrolment n=36**

**Appendix B1: Kaplan-Meier Survival Estimate of Stroke Survivors**

**Appendix B2: Kaplan-Meier Survival Estimates of Survivors by Cognitive Function**

Appendix B3: Hazard Function from the Weibull Model


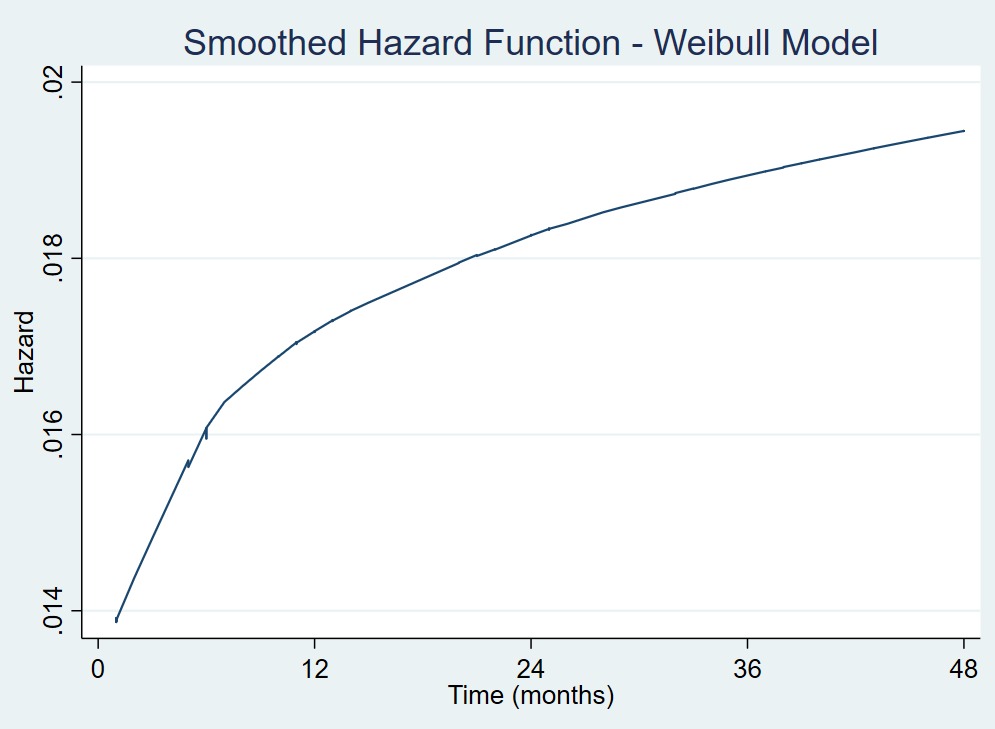


Appendix C: Tables of Results
